# Microbiomic insights into the unique effects of vaginal microbiota on preterm birth in Chinese pregnant women

**DOI:** 10.3389/fmicb.2025.1560528

**Published:** 2025-03-17

**Authors:** Jun Zhang, Zhimin Xu, Mengjun Zhang, Jiaoning Fang, Yijing Zheng, Caihong Jiang, Mian Pan

**Affiliations:** ^1^Department of Obstetrics and Gynecology, Fujian Maternity and Child Health Hospital, College of Clinical Medicine for Obstetrics and Gynecology and Pediatrics, Fujian Medical University, Fuzhou, China; ^2^Department of Medical Ultrasonics, Fujian Maternity and Child Health Hospital, College of Clinical Medicine for Obstetrics and Gynecology and Pediatrics, Fujian Medical University, Fuzhou, China

**Keywords:** preterm birth, *α*-diversity, pathway analysis, prediction model, verification

## Abstract

Preterm birth is a major cause of perinatal morbidity and mortality. The disruption of vaginal microbiota in pregnant women is the most significant risk factor for preterm delivery. In this study, 65 pregnant women were enrolled, of which 29 were women with term births and 36 were women with preterm births, and were then categorized based on gestational age at delivery. The results showed that the *α*-diversity (ACE, Chao1, Simpson, and Shannon indices) of the vaginal microbiota in the term birth group (TG) was significantly higher than that in the preterm birth group (PG). The relative abundance of beneficial bacteria (e.g., *Lactobacillus*) was significantly reduced in the PG compared to the TG, while the relative abundance of harmful bacteria (e.g., *Gardnerella*, *Atopobium*, *Ralstonia*, and *Sneathia*) was significantly increased. A prediction model for gestational age at delivery was established based on key microbial phylotypes, and this model was further verified using clinical samples. Statistical analysis revealed that the prediction model utilizing *Methyloversatilis*, *Atopobium*, *Ralstonia*, *Sneathia*, *Brevundimonas*, *Gardnerella*, *Acinetobacter*, and *Peptostreptococcus* had higher accuracy. These results suggest that certain bacteria could serve as prospective predictors for preterm birth and provide a theoretical basis for the treatment of preterm birth.

## 1 Introduction

Preterm birth is defined as any birth that occurs before 37 weeks of gestation (or fewer than 259 days from the first day of a woman’s last menstrual period), including both spontaneous and iatrogenic preterm births. Preterm birth is a major cause of infant morbidity and mortality worldwide and is strongly associated with long-term adverse outcomes in children (Xie et al., 2022). The high incidence of preterm birth not only increases the annual societal economic burden but also seriously impacts family happiness and social harmony. According to the Global Disease Burden Study, over 15 million infants are born prematurely every year, and the prevalence of preterm birth is continuing to rise (Adane et al., 2024). Among these preterm infants worldwide, approximately 45% of them are diagnosed with spontaneous preterm labor with intact membranes, while approximately 30% are diagnosed with spontaneous preterm labor with ruptured membranes (Beernink et al., 2023). According to a previous report, the occurrence of preterm birth is associated with risk factors such as a short cervix, geographical location, extremes of maternal age (<25 years and >35 years) and body mass index (BMI < 18 and BMI > 28), low socioeconomic status, smoking, and genetic polymorphisms (Wei et al., 2024). For example, it is reported that more than 80% of global preterm births occur in low- and middle-income countries, such as Southern Asia and sub-Saharan Africa (Ohuma et al., 2023). Currently, physical and biochemical markers are commonly used to evaluate preterm pregnancy outcomes; however, they have limited accuracy in predicting its future incidence (Seyama et al., 2022). Therefore, it is necessary to explore effective approaches for identifying preterm birth, which would help reduce the risk of preterm birth and its associated complications.

The vaginal microbiota accounts for 9% of the total human microbiota and plays the most important role in maintaining vaginal homeostasis in women (Golob et al., 2024). The composition of the vaginal microbiota is generally consistent among women of reproductive age and can be classified into five main community state types based on the relative abundance of *Lactobacillus* spp. (Peelen et al., 2019). *Lactobacillus* species are among the major bacteria in the vagina and are widely considered hallmarks of vaginal health, particularly during the reproductive years. A high relative abundance of *Lactobacillus* in the vagina is beneficial for suppressing the growth of harmful bacteria and for elevating the level of short-chain fatty acids, which provide nutrients to support the growth of vaginal epithelial cells (Hong et al., 2021). Some studies have also shown that elevating the abundance of *Lactobacillus* effectively suppresses the production of pro-inflammatory cytokines, alleviates oxidative stress, and regulates vaginal microbiota composition (Chee et al., 2020). On the contrary, a reduction in *Lactobacillus* species and an increase in microbial diversity elevate the risk of bacterial vaginosis, which may be associated with a higher rate of preterm birth. However, this previous study primarily focused on pregnant women in the United States (Fettweis et al., 2019). A previous study also found that the occurrence of preterm birth was significantly higher in pregnant women of Caucasian and Asian descent, associated with decreased *Lactobacillus* abundance and increased *Gardnerella* abundance. However, the *α*/*β*-diversity of the vaginal microbiota in preterm and term births in pregnant women was not analyzed (Callahan et al., 2017). Nevertheless, the combined effect of beneficial bacteria and harmful bacteria with pregnancy outcomes have been rarely studied in the context of vaginal microbiota composition, and the relationship between alterations in the vaginal microbiota and pregnancy outcomes in Chinese women remains unexplored.

This study aimed to identify the characteristic vaginal microbiota in Chinese pregnant women who have preterm birth and to establish a linear relationship between this microbiota and gestational age at delivery. The linear relationship was further verified using clinical samples. These results offer useful information for predicting preterm birth and developing new therapeutics for pregnant women at risk of preterm birth.

## 2 Materials and methods

### 2.1 Recruitment of participants

In the present study, participants were recruited from Fujian Maternity and Child Health Hospital (Fuzhou, China) between January 2021 and June 2023, and the study was approved by the Ethics Committee of Fujian Maternity and Child Health Hospital (Approval no. 2021KLR601). A total of 132 participants—who had not used antibiotics, engaged in sexual activity, used tobacco in the 12 weeks prior to the study, and whose gestational age was 22 ± 2 weeks—provided written informed consent before enrollment in accordance with the approved institutional guidelines. However, only 66 participants met the study requirements, excluding 43 cases of multiple pregnancies, 20 cases of lost contact, two cases of uterine malformation, and one case of severe fetal malformation.

### 2.2 Data collection

The current and historical pregnancy outcomes of the participants were collected and recorded, including maternal age, body mass index (BMI), and the frequency of abortions and fertility. All participants were assigned to two groups based on their pregnancy outcome: the preterm birth group (PG) and the term birth group (TG).

### 2.3 Sample collection

The vagina was examined using a single-use sterile endoscope, and a cotton swab was gently rotated across the vaginal wall for 20 s. The sampling loop was then removed from the cannula and fully inserted into the uterus. The handle of the sampler was rotated 10 times to collect the endometrial sample. During the passage of the sampler through the vagina, the sampling loop remained retracted within the cannula to avoid contact with microorganisms from the cervix and the vagina, thereby eliminating cross-contamination between the intrauterine and vaginal samples. All samples were immediately frozen for 3–4 min using liquid nitrogen and then stored at −80°C until further use. Specific surgical steps and preoperative management for cervical cerclage surgery were carried out according to our previous study (Fang et al., 2020).

### 2.4 Vaginal microbiome composition analysis

Sequencing analysis of the vaginal microbiota was performed using the MiSeq platform, following the methodology described in a previous report with minor modifications (Guo et al., 2021). Briefly, total microbial DNA was extracted from the cervical–vaginal fluid (CVF) samples using a commercially available DNA extraction kit (MoBio, Carlsbad, CA, USA). The V3–V4 regions of bacterial 16S rRNA genes were then amplified using broad-range bacterial primers, namely 338F primers (5′-CCTAYGGGRBGCASCAG-3′) and 806R primers (5′-GGACTACHVGGGTWTCTAAT-3′). These products were purified using 2.0% agarose gel electrophoresis, and the target fragment was collected and recovered using the Agencourt AMPure XP kit (Hangzhou, China). The DNA content of the 65 cases was measured using a NanoDrop ND-2000 spectrophotometer (NanoDrop Technologies, Wilmington, DE, USA). The sequencing libraries consisted of equal concentrations of each sample, and their quality was further evaluated using the Qubit@ 2.0 Fluorometer (Thermo Scientific, CA, USA). The libraries were then sequenced on the Illumina MiSeq platform (San Diego, CA, USA) at Shanghai Biotree Biotech. Co., Ltd.

### 2.5 Vaginal microbiota sequence analysis

After sequencing, raw data were filtered, denoised, and merged. Then, chimeras were removed using Microbial Ecology software (v 2.0). The high-quality sequences were collected and then grouped into operational taxonomic units (OTUs) with over 97% similarity. Taxonomy annotation of the OTU sequences was performed using the mothur package and the SSU rRNA database from SILVA138.1. The *α*/*β*-diversity of the vaginal microbiota were calculated using Xshell (v 7.0). Principal component analysis (PCA), principal coordinates analysis (PCoA), and non-metric multidimensional scaling (NMDS) of the vaginal microbiota from all samples, based on the Bray–Curtis distance, were performed using R software (v 4.4.2). The key microbial phylotypes were screened using Microbial Ecology software. The raw data were stored in the NCBI database (no. PRJNA1122359).

### 2.6 Verification experiment

Participants were recruited from Fujian Maternity and Child Health Hospital (Fuzhou, China) between September 2023 and March 2024. The study was approved by the Ethics Committee of Fujian Maternity and Child Health Hospital (Approval no. 2023KLR936). A total of 12 participants—who had not used antibiotics, engaged in sexual activity, and used tobacco in the 12 weeks prior to the study, and whose gestational age was 22 ± 2 weeks—provided written informed consent before enrollment in accordance with the approved institutional guidelines.

### 2.7 Statistical analysis

All data were presented as mean ± SD. Significant differences were evaluated using Welch’s *t*-test with GraphPad Prism (version 9.0).

## 3 Results

### 3.1 Clinical characteristics and pregnancy outcomes of the participants

As indicated in Table 1, a total of 66 participants were recruited, namely 29 pregnant women with term birth (accounted for 43.94%) and 37 pregnant women with preterm birth (accounted for 56.06%). There were no significant differences in maternal age, BMI, gravida, parity, and cervical length between pregnant women in the PG and those in the TG (*p* > 0.05). However, the gestational age at delivery of pregnant women in the PG (32.27 ± 3.29 weeks) was lower than that of pregnant women in the TG (38.16 ± 1.03 weeks) (*p* < 0.05). Similarly, the body weight of infants in the PG (2007.93 ± 559.58 g) was significantly lower than that of infants in the TG (3188.07 ± 435.89 g) (*p* < 0.05).

**Table 1:** Descriptive statistics of the study participants.

Characteristic	PG (*n* = 37)	TG (*n* = 29)	*P*-value
Age (years)	31.19 (3.68)	30.6 5 (3.58)	0.56
BMI (m^2^/kg)	23.85 (3.54)	22.79 (3.34)	0.22
Gravida	3.0 (1.44)	2.29 (1.01)	0.057
Parity	0.56 (0.67)	0.42 (0.56)	0.36
Cervical length (cm)	1.34 (0.67)	1.70 (1.17)	0.24
Gestational age at delivery (weeks)	32.27 (3.29)	38.16 (1.03)	5.90 × 10^−11^
Birth weight (g)	2007.93 (559.58)	3188.07 (435.89)	3.50 × 10^−12^

Data are presented as mean ± SD.

### 3.2 Alteration in the *α*-diversity of vaginal microbiota in pregnant women with preterm birth

**Figure 1:**
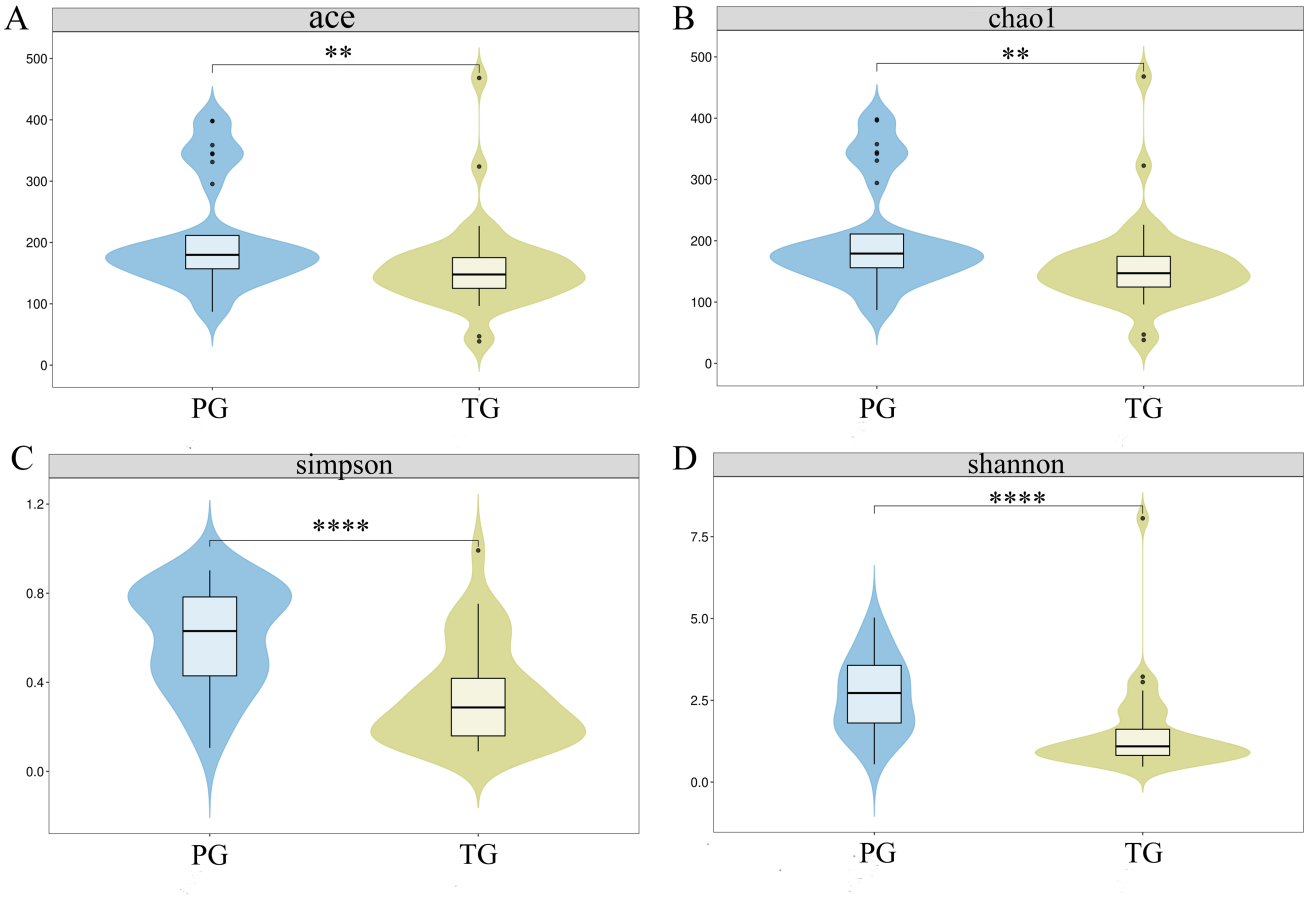
Alterations in the *α*-diversity of the vaginal microbiota in pregnant women with preterm birth. **(A)** ACE index; **(B)** Chao1 index; **(C)** Simpson index, and **(D)** Shannon index. *p < 0.05, **p < 0.01, ***p < 0.001, and ****p < 0.0001, respectively.

The ACE, Chao1, Simpson, and Shannon indices are the most important parameters in *α*-diversity analysis for assessing the diversity of species in various environments, such as soil, the gut, and the vagina. As shown in Figure 1, the ACE (209.30 ± 88.43), Chao1 (208.65 ± 88.21), Simpson (0.60 ± 0.23), and Shannon (2.63 ± 1.16) indices of the vaginal microbiota in the PG were significantly higher than those in the TG (159.16 ± 72.44, 158.49 ± 72.36, 0.33 ± 0.22, and 1.49 ± 1.32, respectively) (*p* < 0.01), suggesting that the changes in the *α*-diversity of the vaginal microbiota may serve as important parameters for predicting the risk of preterm birth.

### 3.3 Alteration in the vaginal microbiota of pregnant women with preterm birth

**Figure 2:**
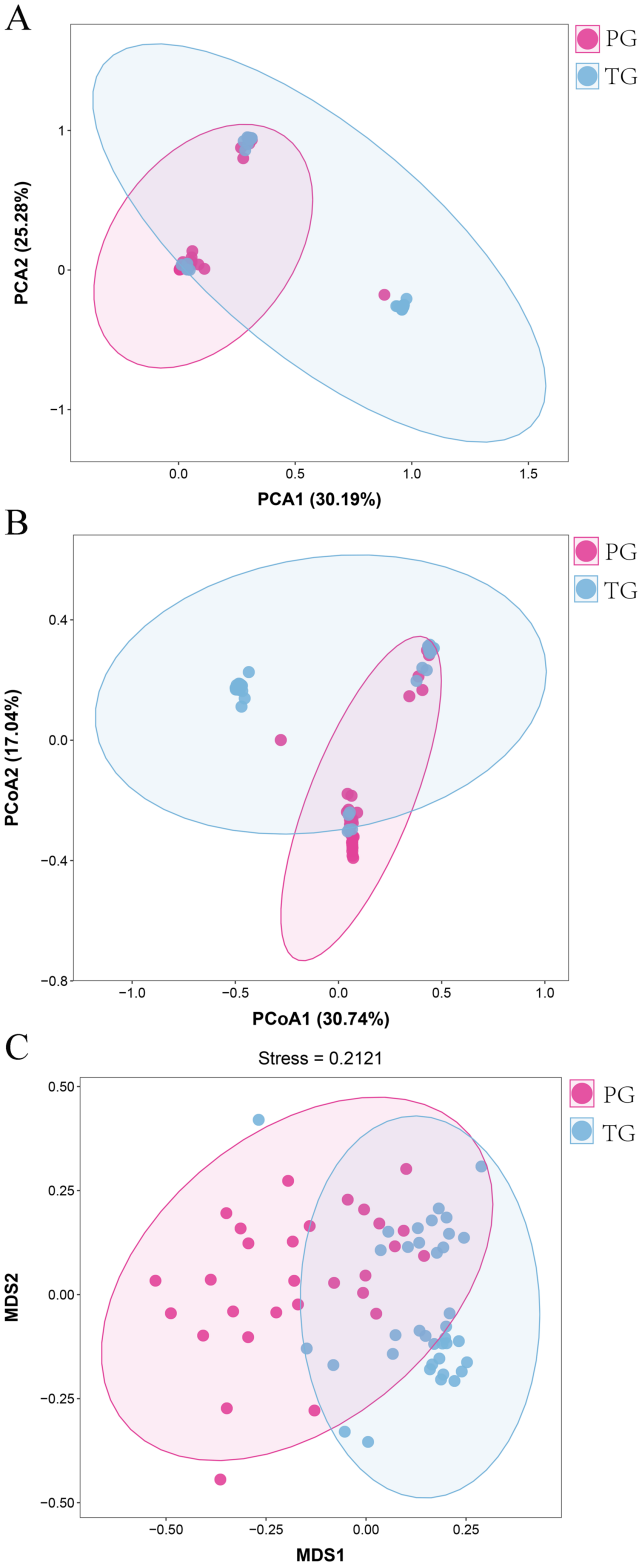
Overall alterations in the vaginal microbiota in pregnant women with preterm birth. **(A)** PCA; **(B)** PCoA, and **(C)** NMDS analysis.

The *β*-diversity intuitively reflects the similarities in vaginal microbiota composition between different groups. Therefore, PCA, PCoA, and NMDS were used to reveal the differences in the vaginal microbiota composition of pregnant women between the PG and TG. As shown in Figure 2A, the first and second principal components accounted for 30.19 and 25.28% of the total variation in the PCA score plot based on the Bray–Curtis distance, respectively. There were clear differences in the vaginal microbiota of pregnant women between the PG and TG, indicating that the vaginal microbiota composition was significantly altered in pregnant women in the PG compared to those in the TG. PCoA is a chemometric technique that fundamentally simplifies data sets by projecting them into a space defined by a small number of orthogonal axes. As shown in Figure 2B, the PCoA score plot showed a clear clustering of vaginal microbiota composition between the PG and TG. In addition, the result of the NMDS analysis further confirmed the findings of PCA and PCoA (Figure 2C).

**Figure 3:**
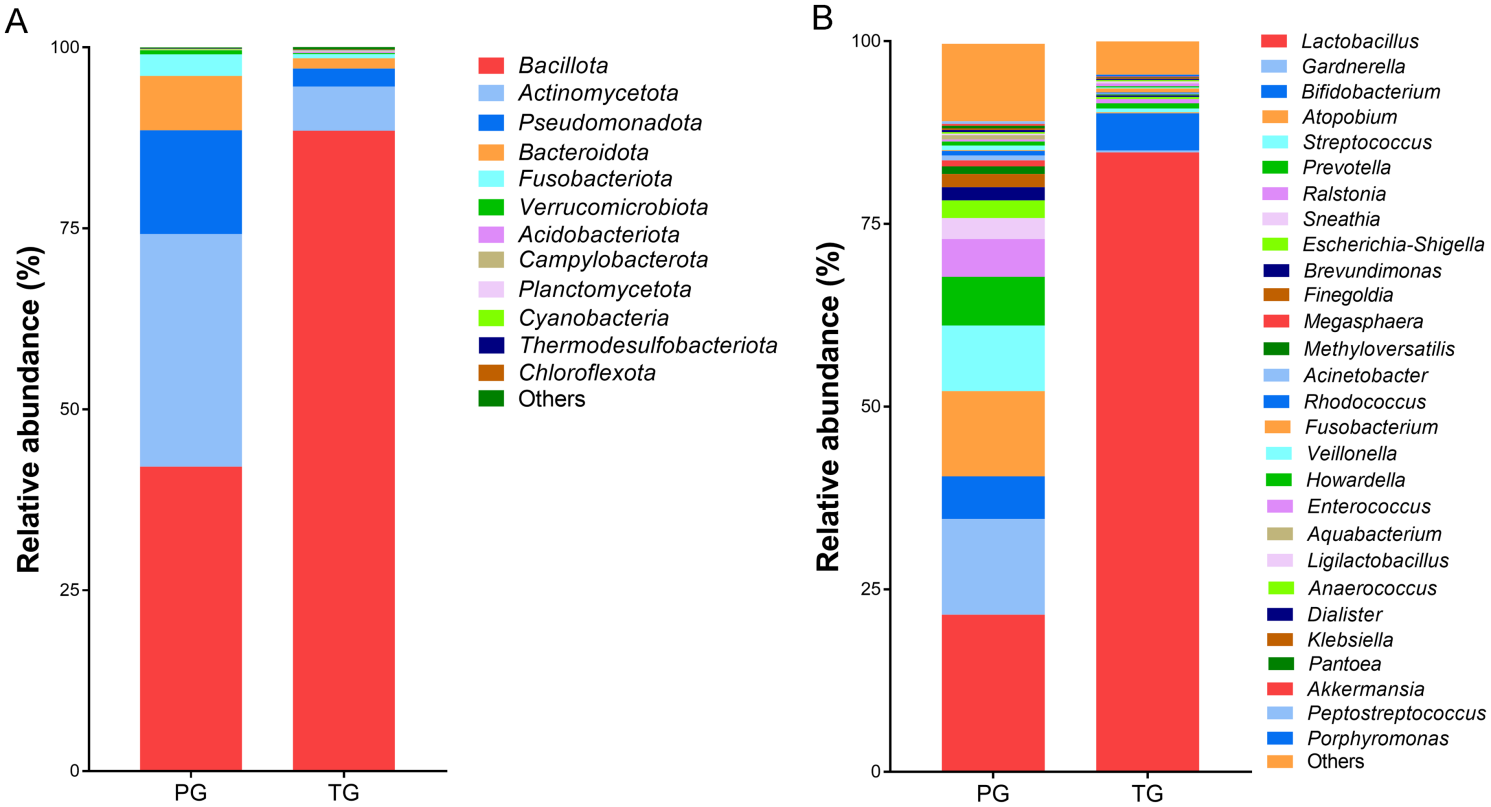
The percentage of community abundance at **(A)** the phylum level and **(B)** the genus level.

Further analysis revealed altered vaginal microbiota composition at different levels. As shown in Figure 3A, Bacillota, Actinomycetota, Campylobacterota, Bacteroidota, Fusobacteriota, and Verrucomicrobiota exhibited higher relative abundance in pregnant women from the PG and TG at the phylum level. Among these, Bacillota accounted for 42.07% of the vaginal microbiota in the PG and 88.47% in the TG. Actinomycetota was the second most abundant phylum, comprising 32.11% of the vaginal microbiota in the PG and 6.12% in the TG. In addition, *Lactobacillus*, *Gardnerella*, *Bifidobacterium*, *Atopobium*, *Streptococcus*, *Prevotella*, *Ralstonia*, *Sneathia*, and *Escherichia*-*Shigella* were the predominant bacteria at the genus level (Figure 3B). Among these, *Lactobacillus* was the most predominant genus, accounting for 21.51% of the vaginal microbiota in the PG and 84.76% in the TG. *Gardnerella* was the second most abundant genus, which accounted for 13.09% of the vaginal microbiota in the PG and 0.26% in the TG. In addition, *Bifidobacterium* was also one of the most abundant genera, accounting for 5.86% of the vaginal microbiota in the PG and 5.09% in the TG.

### 3.4 Screening for key vaginal microbial phylotypes in pregnant women of the PG

**Figure 4:**
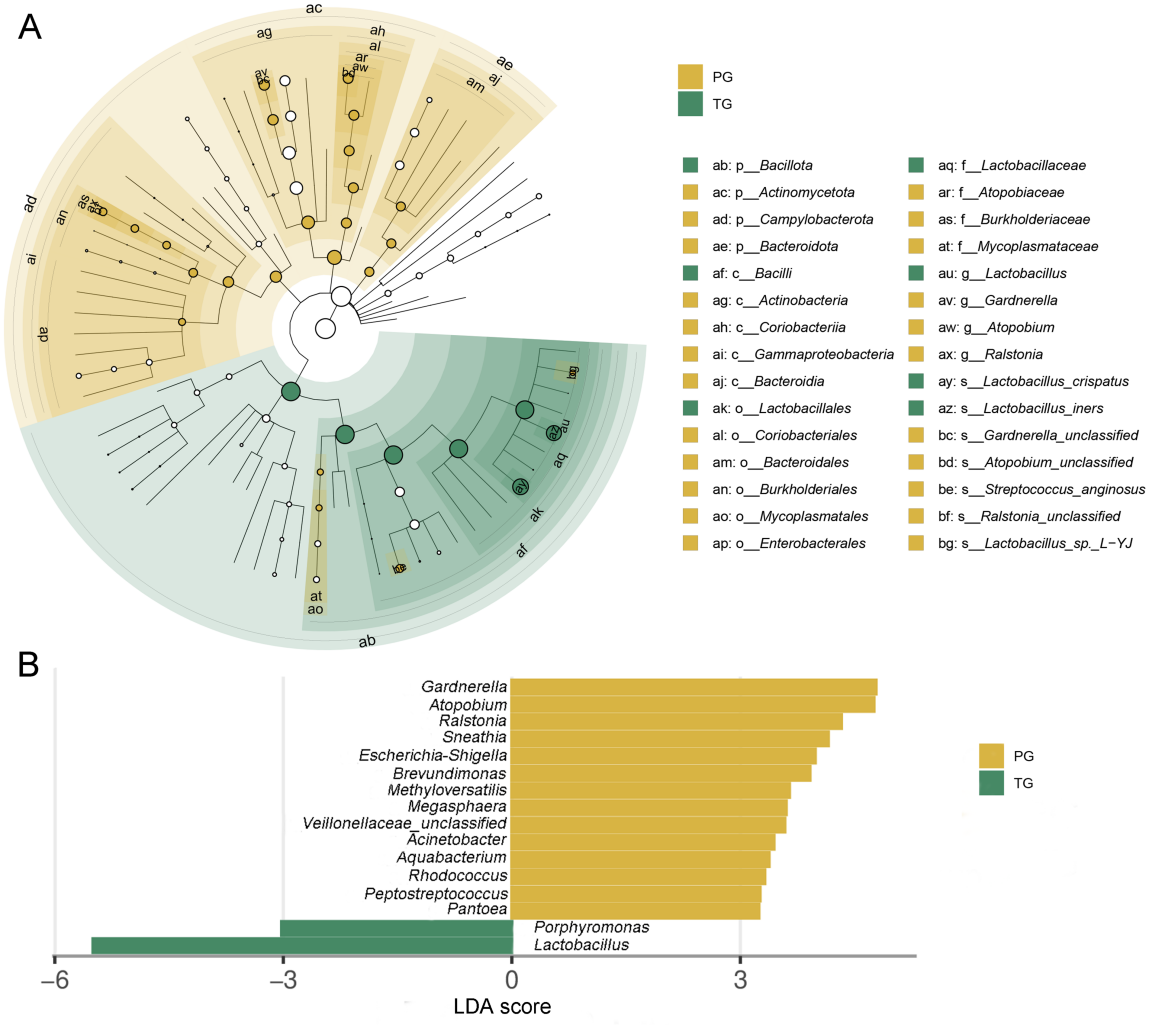
Linear discriminant analysis effect size (LEfSe) analysis of the vaginal microbiota in pregnant women. **(A)** Taxonomic cladogram produced from the LEfSe analysis; **(B)** histogram of the LDA scores (>3.0 in both cases).

The linear discriminant analysis effect size (LEfSe) analysis, based on an LDA score <3.0, found that the relative abundance of Bacillota at the phylum level was significantly reduced among pregnant women in the PG compared to those in the TG (*p* < 0.05). However, the relative abundance of Actinomycetota, Campylobacterota, and Bacteroidota was significantly increased (*p* < 0.05) (Figure 4A). In addition, the relative abundance of *Lactobacillus* and *Porphyromonas* at the genus level was significantly reduced among pregnant women in the PG compared to those in the TG. However, the relative abundance of *Gardnerella*, *Atopobium*, *Ralstonia*, *Sneathia*, *Escherichia*-*Shigella*, *Brevundimonas*, *Methyloversatilis*, *Megasphaera*, *Acinetobacter*, *Aquabacterium*, *Rhodococcus*, and *Peptostreptococcus* was significantly increased (Figure 4B). The above results indicated that the vaginal microbiota composition was significantly altered among pregnant women in the PG, which may be one of the key factors contributing to preterm birth.

**Figure 5:**
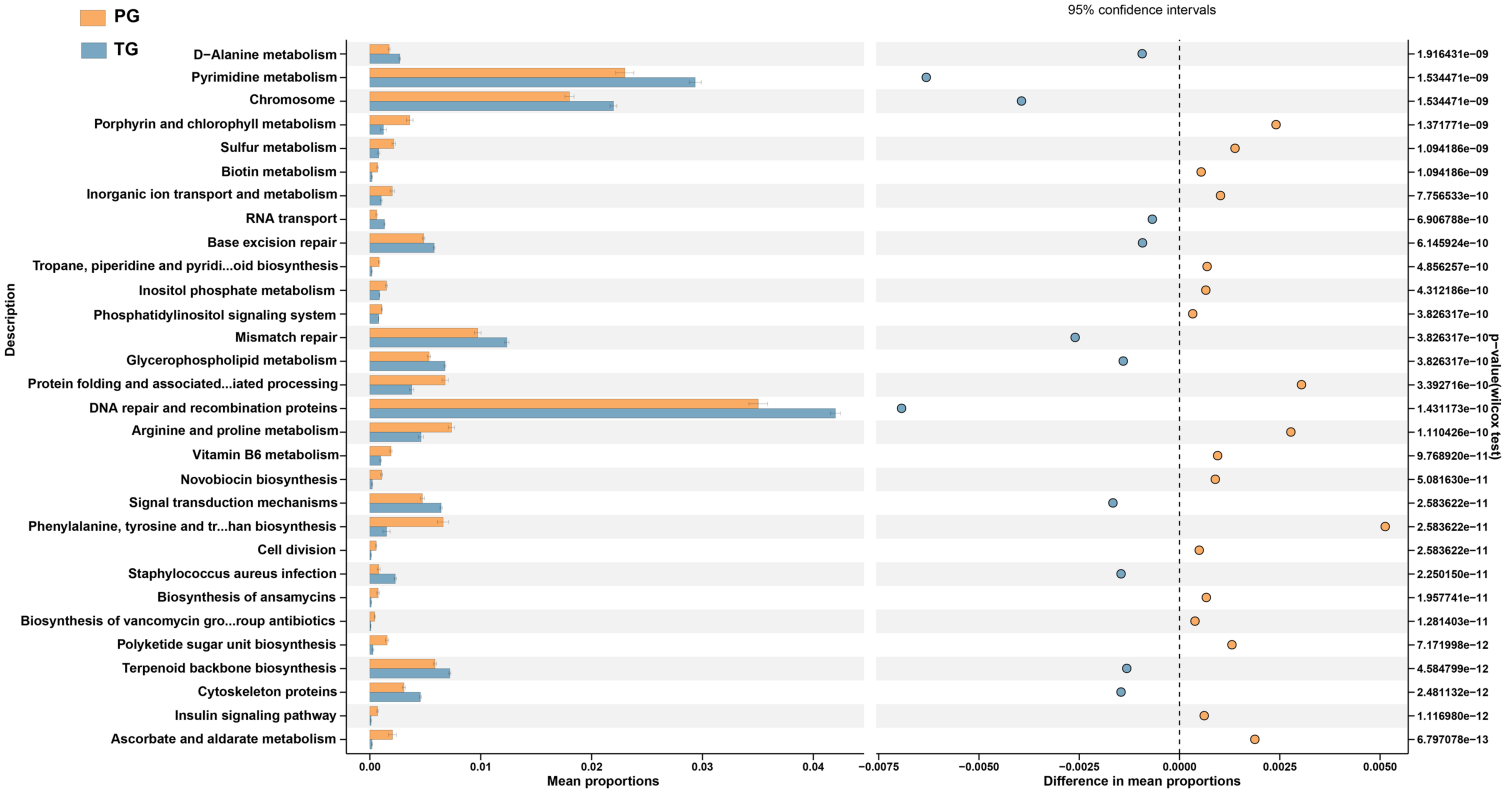
STAMP analysis of PICRUSt based on the KEGG pathway analysis (KEGG level 3) in pregnant women with preterm birth and term birth.

To further identify the alterations in pathways related to the differential vaginal microbiota of pregnant women in the PG, STAMP analysis were applied to identify differentially altered pathways based on the differential vaginal microbiota. As shown in Figure 5, D-Alanine metabolism, pyrimidine metabolism, chromosomes, RNA transport, base excision repair, mismatch repair, glycerophospholipid metabolism, DNA repair and recombination proteins, signal transduction mechanisms, *staphylococcus aureus* infection, terpenoid backbone biosynthesis, and cytoskeleton proteins were significantly downregulated among pregnant women in the PG compared to those in the TG (*p* < 0.05). However, porphyrin and chlorophyll metabolism; sulfur metabolism; biotin metabolism; inorganic ion transport and metabolism; tropane, piperidine, and pyridine alkaloid biosynthesis; inositol phosphate metabolism; phosphatidylinositol signaling system; protein folding and associated processing; arginine and proline metabolism; vitamin B6 metabolism; novobiocin biosynthesis; cell division; biosynthesis of ansamycins; biosynthesis of vancomycin group antibiotics; polyketide sugar unit biosynthesis; insulin signaling pathway; and ascorbate and aldarate metabolism were significantly upregulated (*p* < 0.05).

### 3.5 Association between key microbial phylotypes and gestational age at delivery

**Figure 6:**
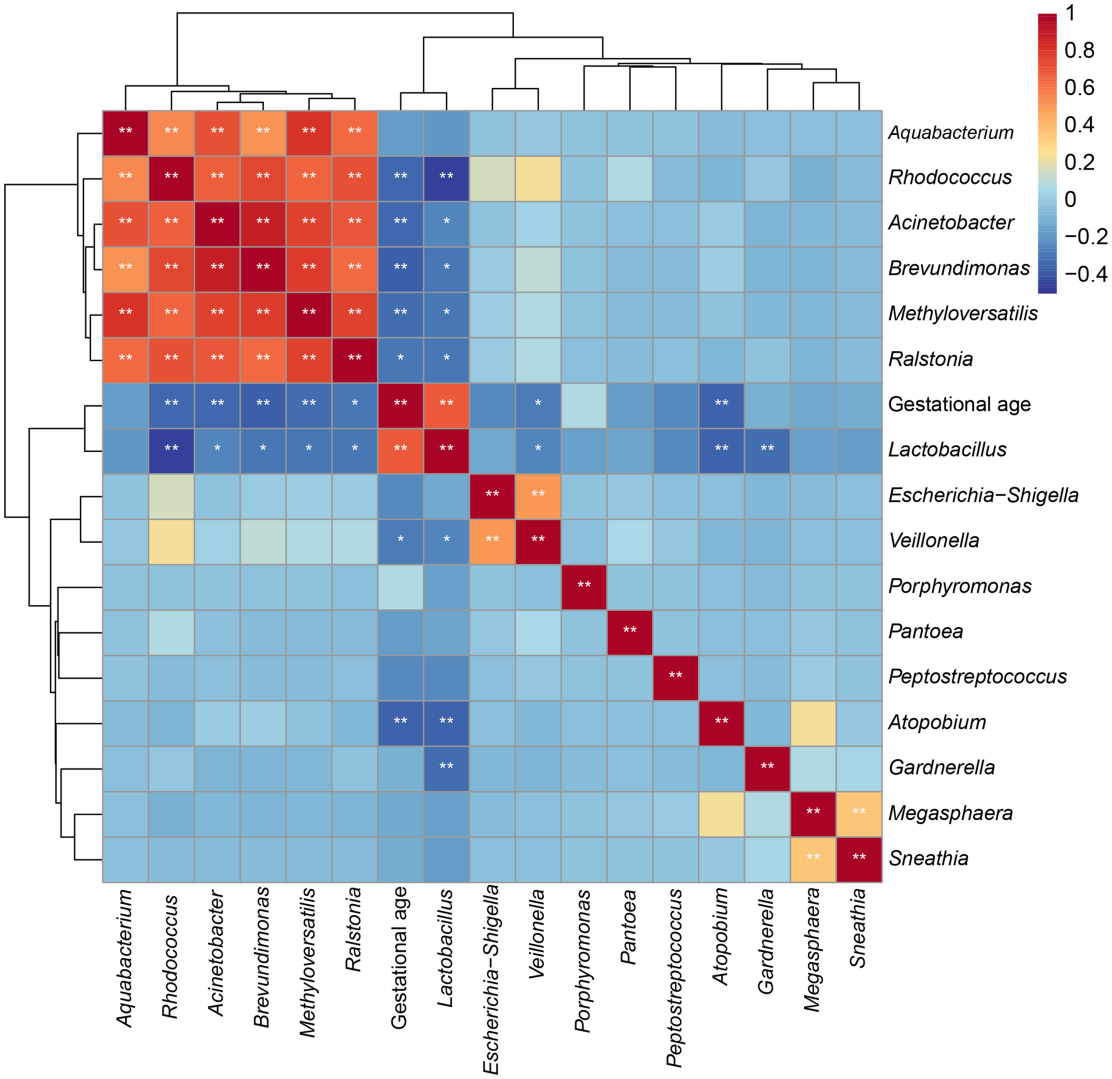
Coefficient matrix heatmap showing the correlation between the key vaginal microbiota and gestational age at delivery.

To identify the association between alterations in the vaginal microbiota and gestational age at delivery, we further evaluated the correlation between the abovementioned altered microbial genera and gestational age. As shown in Figure 6 and Supplementary Figure S1, gestational age was positively related to the relative abundance of *Lactobacillus* (y = 6.147x − 165.3, R^2^ = 0.4724, and *p* < 0.0001) and *Porphyromonas* (y = 0.01681x – 0.4647, R^2^ = 0.005793, and *p* = 0.5106) but it was negatively related to the relative abundance of *Methyloversatilis* (y = −0.09723x + 4.043, R^2^ = 0.1099, and *p* = 0.0065), *Atopobium* (y = −1.453 + 57.77, R^2^ = 0.1306, and *p* = 0.0029), *Ralstonia* (y = −0.4204x +17.79, R^2^ = 0.09089, and *p* = 0.0139), *Sneathia* (y = −0.211x + 8.9, R^2^ = 0.01253, and *p* = 0.3708), *Escherichia*-*Shigella* (y = −0.2672x +10.88, R^2^ = 0.05233, and *p* = 0.0647), *Brevundimonas* (y = −0.1741x + 7.20, R^2^ = 0.1423, and *p* = 0.0018), *Gardnerella* (y = −0.419x +21.05, R^2^ = 0.01095, and *p* = 0.4031), *Megasphaera* (y = −0.05033x + 2.189, R^2^ = 0.0176, and *p* = 0.2883), *Acinetobacter* (y = −0.06524x +2.725, R^2^ = 0.1186, and *p* = 0.0046), *Rhodococcus* (y = −0.03978x + 1.797, R^2^ = 0.114, and *p* = 0.0056), *Veillonella* (y = −0.08165x + 3.3, R^2^ = 0.07887, and *p* = 0.0224), *Aquabacterium* (y = −0.05172x + 2.156, R^2^ = 0.03133, and *p* = 0.1551), *Pantoea* (y = 0.05148x + 2.061, R^2^ = 0.03289, and *p* = 0.1450), and *Peptostreptococcus* (y = −0.04222x + 1.684, R^2^ = 0.05161, and *p* = 0.5106).

### 3.6 Validation of linear relationships

**Figure 7:**
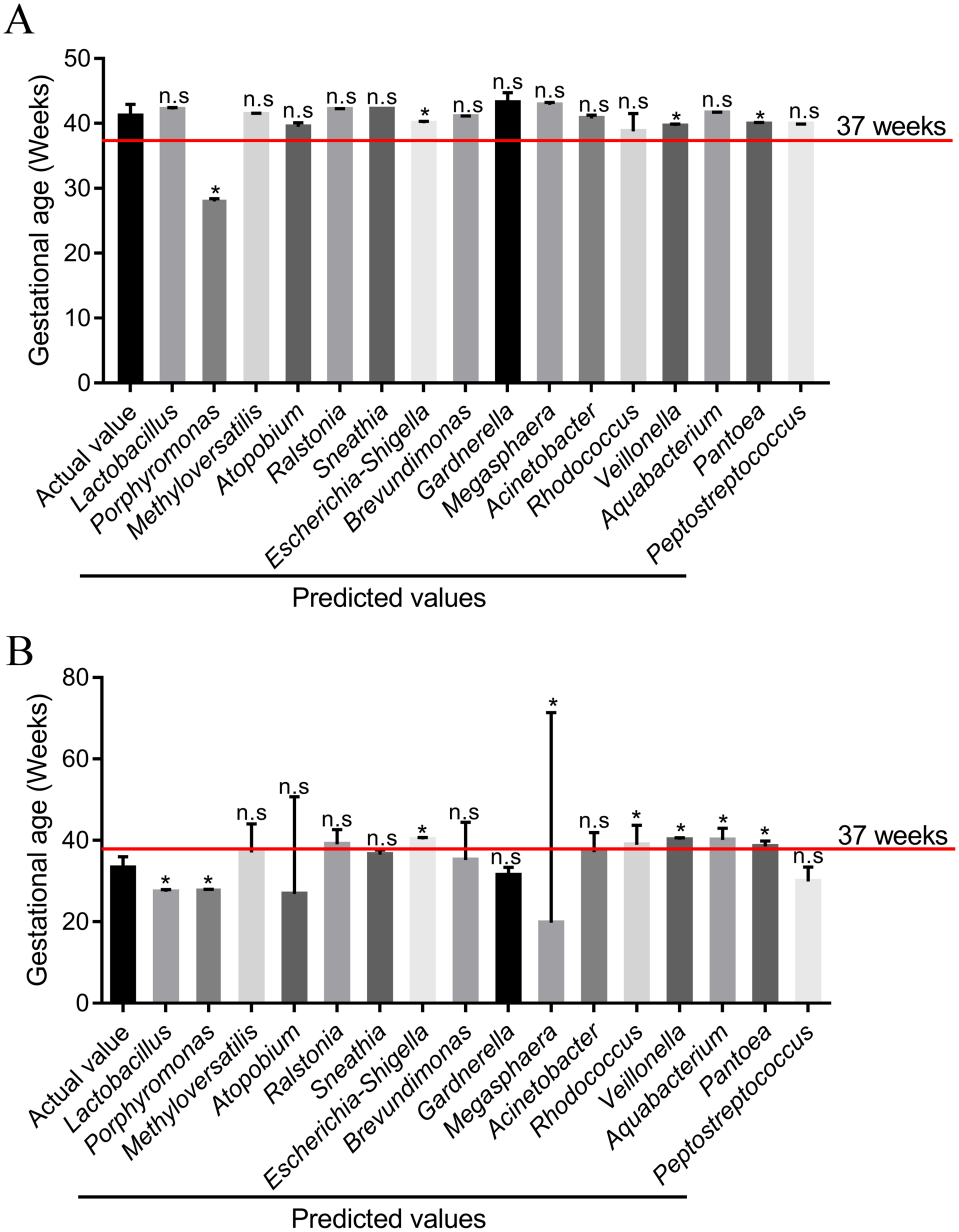
Comparison of the predicted values of the responses from the model and the experimental values (*n* = 12). **(A)** Pregnant women with term birth (*n* = 7); **(B)** pregnant women with preterm birth (*n* = 5). *p < 0.05 and **p < 0.01, respectively, and n.s represents a *p*-value more than 0.05.

To further explore the validity of the model equation in predicting gestational age, 12 participants were recruited. According to their pregnancy outcomes, the 12 participants consisted of seven cases of pregnancy women with term birth and five cases of pregnancy women with preterm birth. Among pregnant women in the TG group, there were no significant differences between the actual and predictive values for *Lactobacillus*, *Methyloversatilis*, *Atopobium*, *Ralstonia, Sneathia*, *Brevundimonas*, *Gardnerella*, *Megasphaera*, *Acinetobacter*, *Rhodococcus, Aquabacterium*, and *Peptostreptococcus* (*p* > 0.05) (Figure 7 and Supplementary Table S1). Among pregnant women in TG, there was no significant difference in *Methyloversatilis*, *Atopobium*, *Ralstonia*, *Sneathia*, *Brevundimonas*, *Gardnerella*, *Acinetobacter*, and *Peptostreptococcus* between the actual and predictive values (*p* > 0.05). The findings of the study reveal that the abundance of *Methyloversatilis*, *Atopobium*, *Ralstonia*, *Sneathia*, *Brevundimonas*, *Gardnerella*, *Acinetobacter*, and *Peptostreptococcus* can be used to effectively predict gestational age at delivery.

## 4 Discussion

Preterm birth has become an alarming public health concern because of the relatively higher mortality rate among preterm infants. According to a previous report, approximately three-quarters of preterm birth cases are diagnosed as spontaneous, which includes those with a history of spontaneous preterm birth or preterm pre-labor rupture of membranes (Humberg et al., 2020). Despite significant advancements in detection methods, accurately predicting gestational age at delivery remains challenging, and this challenge is particularly critical because gestational age is a major determinant of mortality in premature infants (Park et al., 2022). With the development of science and technology, the gut, vaginal, and skin microbiota have drawn widespread attention due to their vital role in improving host health. Therefore, we hypothesized that preterm birth is related to alterations in the vaginal microbiota and its metabolites. In the present study, 16S rRNA gene sequencing and multiple statistical analyses were used to detect and identify the vaginal microbiota. In addition, we found a potential association between key microbial phylotypes and gestational age at delivery, which suggests that these phylotypes can be used to predict gestational age at delivery.

Some studies have shown that the risk of preterm birth is strongly related to the BMI of pregnant women. Specifically, a BMI of more than 28.0 or <18.0 elevates the risk of preterm birth (Cnattingius et al., 2013). For example, a high BMI in pregnant women increases the risk of gestational diabetes, hypertensive conditions, and fetal malformations, which are among the most common causes of medically indicated preterm birth (Robinson et al., 2021). In addition, both advanced maternal age (more than 35 years) and younger maternal age (<25 years) further increase the risk of preterm birth, which may be associated with low birth weight in preterm infants and severe neonatal conditions (Wu et al., 2024). Therefore, participants were selected based on a BMI range of 18–28 and an age range of 25–35 years in this study. This approach helps eliminate the influence of BMI and maternal age on the risk of preterm birth. The body weight of preterm infants was significantly lower than that of term infants due to the incomplete development of preterm infants. This observation is in agreement with the result of a previous study (Kozuki et al., 2015). In addition, there is a significant difference in vaginal microbiota composition across different stages of pregnancy (McCauley et al., 2019). Therefore, samples were collected at the 22nd week of pregnancy (±2 weeks), which helps minimize the influence of other factors.

Currently, the study of vaginal microbiota is relatively limited compared to that of the gut microbiota. However, the vaginal microbiota accounts for approximately 9% of the total human microbiota and plays an essential role in improving vaginal health. Vaginal microbiota is a dynamic ecosystem composed of various microorganisms in different quantities and ratios. This ecosystem helps maintain the integrity of the vaginal barrier and prevents the growth of harmful bacteria (Shen et al., 2016). The *α*-diversity of the vaginal microbiota in healthy women is significantly lower than that in women with vaginal diseases, which differs from the pattern observed in the gut microbiota (Ou et al., 2024). In the present study, the ACE, Chao1, Simpson, and Shannon indices of vaginal microbiota were significantly elevated in the PG, which is in agreement with a previous report (Karambelkar and Baranwal, 2024). In addition, the α-diversity of vaginal microbiota is frequently used to establish prediction models for gestational age at delivery (Haque et al., 2017). However, the α-diversity of vaginal microbiota is clearly altered during pregnancy, which significantly interferes with the accuracy of these prediction models.

It is well known that the relative abundance of *Lactobacillus* is significantly higher than that of other genera found in reproductive-age women. However, there is a notable difference in the abundance of *Lactobacillus* between pregnant women with preterm birth and those with term birth. Some studies have also reported that pregnant women with preterm birth have a lower relative abundance of *Lactobacillus* in the vagina. Feehily et al. (2020) found that *Lactobacillus* species in the vagina include *L. crispatus*, *L. delbruecki*, *L. gasseri*, *L. gasseria*, *L. H fermentum*, *L. H gastricus*, *L. helveticus*, *L. jensenii*, *L. kefiranofaciens*, and *L. taiwanensis*. *Lactobacillus* is regarded as a beneficial probiotic for host health when taken in adequate amount. It has various physiological effects, including the suppression of oxidative stress and inflammatory responses (Qin et al., 2024). *Lactobacillus* also prevents the growth of harmful bacteria in the vagina by increasing the concentrations of short-chain fatty acids. In this study, the analysis of vaginal microbiota revealed that the relative abundance of *Lactobacillus* was positively correlated with gestational age at delivery. Therefore, *Lactobacillus* supplementation may become an effective method to prevent the occurrence of preterm birth.

*Gardnerella* is commonly present in women of childbearing age, and a high relative abundance of *Gardnerella* can cause a series of diseases. It is also extensively used to establish models of bacterial vaginosis (Jothi et al., 2024). Meanwhile, *Gardnerella* can produce sialidase and proline aminopeptidase that destroy the protective factors in the vagina and facilitate the adhesion of anaerobic bacteria to the vaginal mucosa surface. *Gardnerella* can promote the growth and reproduction of pathogenic bacteria by producing massive amounts of amines, which further elevate the risk of preterm birth (Gao et al., 2021). *Atopobium* is one of the essential genera in the vagina microbiota, but its abundance is positively associated with the incidence rate of infertility, endometritis, and pelvic inflammatory disease (Ravel et al., 2021). *Ralstonia* belongs to the family *Burkholderiaceae* of the class *Betaproteobacteria* and stimulates metabolic inflammation by activating the CD14/TLR4 pathway, which is associated with the production of lipopolysaccharides (Zhang-Sun et al., 2019). *Sneathia* is an anaerobic Gram-negative bacterium that is regarded as an opportunistic pathogen of the reproductive tract that is strongly associated with the occurrence of spontaneous abortions, preterm labor, and postpartum bacteremia (Duployez et al., 2020). *Escherichia*-*Shigella* is widely considered a potentially pathogenic bacterium that is closely related to the occurrence and development of inflammatory responses (Bai et al., 2023). *Brevundimonas* is a human pathogen that primarily causes catheter-associated bacteremia in immunosuppressed patients (Soto et al., 2022). *Acinetobacter* has been known as a menacing bacterial pathogen since the 1970s, causing a wide range of infections, including bloodstream infections, catheter-associated infections, and ventilator-associated infections (Allemailem, 2023). *Peptostreptococcus* is mainly found in the stool and mucosal microbiota of individuals with intestinal diseases. It induces inflammatory responses by upregulating the toll-like receptor (TLR) signaling and AMP-activated protein kinase (AMPK) signaling pathways (Tsoi et al., 2017). In this study, the relative abundance of *Gardnerella*, *Atopobium*, *Ralstonia*, *Sneathia*, *Brevundimonas*, and *Peptostreptococcus* was negatively associated with gestational age at delivery. Therefore, we speculated that reducing the abundance of these bacteria may be beneficial for extending gestational age at delivery.

## 5 Conclusion

In the present study, we found a significant difference in the vaginal microbiota between pregnant women with preterm birth and those with term birth. This difference was characterized by a reduction in beneficial bacteria and an increase in harmful bacteria. In addition, the key microbial phylotypes were screened, which were used to establish a prediction model for gestational age at delivery. However, the accuracy of the prediction model was affected by the quantity of the samples. In future studies, it will be necessary to further optimize this predictive model by collecting more clinical samples and using third-generation sequencing technology.

## Data Availability

The original contributions presented in the study are publicly available. This data can be found at: https://www.ncbi.nlm.nih.gov/, accession number PRJNA1122359.
