# Supplementary material for: Microbiomic insights into the unique effects of vaginal microbiota on preterm birth in Chinese pregnant women
Source: Front Microbiol. 2025 Mar 17;16:1560528. doi: 10.3389/fmicb.2025.1560528 (PMC11955808; doi:10.3389/fmicb.2025.1560528)
Supplement: Supplementary file 1 [file Data_Sheet_1.docx]

**Supplementary data**

**Figure S1** Linear correlation analysis of the key vaginal microbiota and gestational age at delivery.


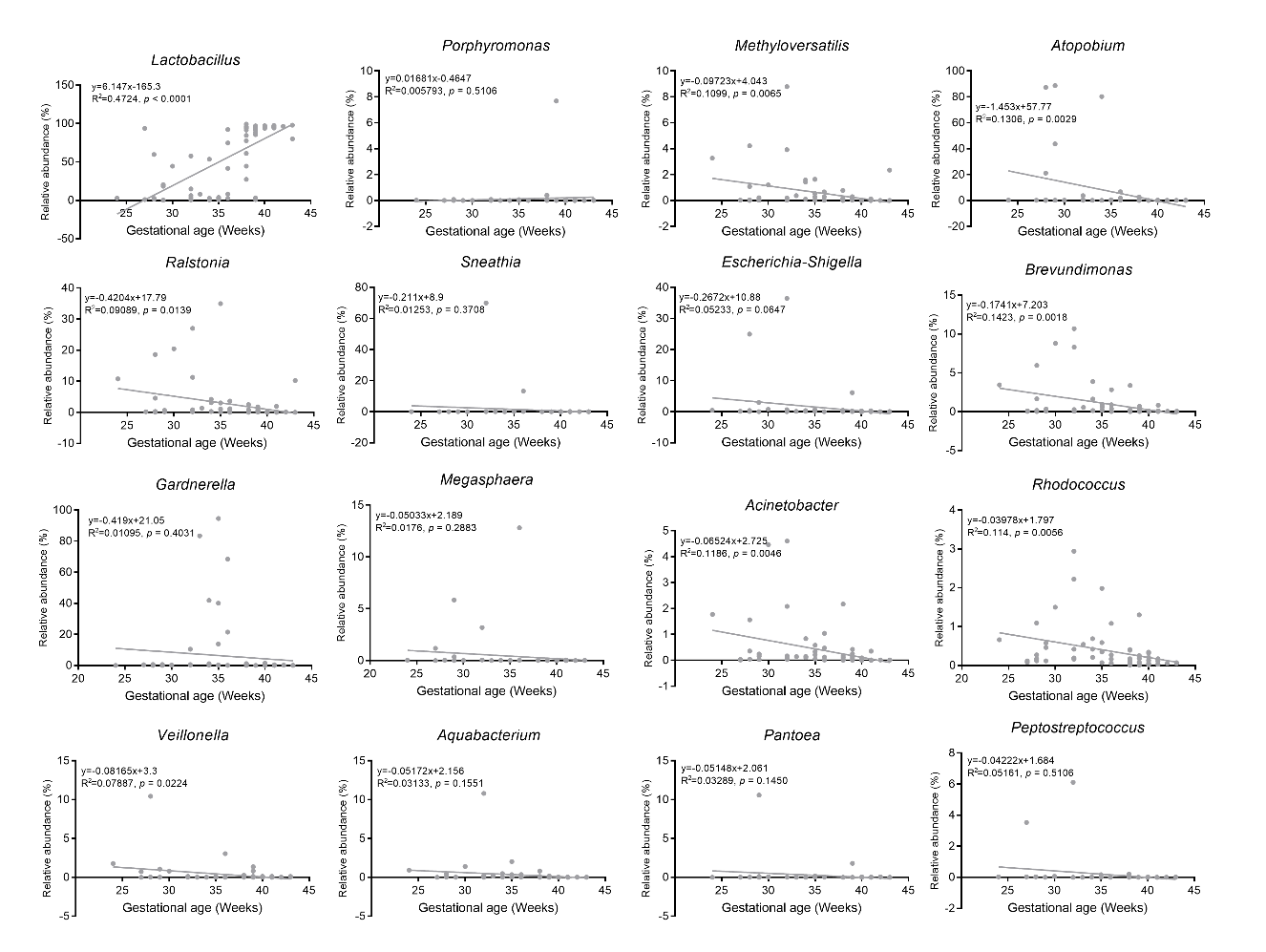


**Table S1** The comparison of predicted values of responses from the model to experimental values (n=12). Data are presented as mean ± SD.

| Genus | Pregnancy women with term birth (n = 7, 41.13±1.81) | | | Pregnancy women with preterm birth (n = 5, 33.20 ± 2.77) | | |
| --- | --- | --- | --- | --- | --- | --- |
|  | Relative abundance/% | Predicted values (weeks) | *p* value | Relative abundance/% | Predicted values (weeks) | *p* value |
| *Lactobacillus* | 94.21 ± 1.80 | 42.16 ± 0.27 | > 0.05 | 2.75 ± 3.29 | 27.34 ± 0.54 | < 0.05 |
| *Porphyromonas* | 0.07 ± 0.17 | 27.97±0.44 | < 0.05 | 0.00 ± 0.00 | 27.75 ± 0.23 | < 0.05 |
| *Methyloversatilis* | 0.02 ± 0.02 | 41.39±0.17 | > 0.05 | 0.44 ± 0.68 | 37.06 ± 6.96 | > 0.05 |
| *Atopobium* | 0.37 ± 0.91 | 39.46±0.66 | > 0.05 | 18.83 ± 34.74 | 26.80 ± 23.91 | > 0.05 |
| *Ralstonia* | 0.07 ± 0.06 | 42.13±0.14 | > 0.05 | 1.40 ± 1.153 | 39.00 ± 3.65 | > 0.05 |
| *Sneathia* | 0.00 ± 0.00 | 42.18±0.00 | > 0.05 | 1.19 ± 0.23 | 36.53 ± 1.11 | > 0.05 |
| *Escherichia*-*Shigella* | 0.20 ± 0.08 | 40.03±0.27 | < 0.05 | 0.14 ± 0.13 | 40.21 ± 0.50 | < 0.05 |
| *Brevundimonas* | 0.05 ± 0.02 | 41.06±0.09 | > 0.05 | 1.08 ± 1.61 | 35.18 ± 9.24 | > 0.05 |
| *Gardnerella* | 2.98 ± 0.60 | 43.19±1.52 | > 0.05 | 7.89 ± 0.80 | 31.42 ± 1.92 | > 0.05 |
| *Megasphaera* | 0.03 ± 0.02 | 42.84±0.39 | > 0.05 | 1.19 ± 2.60 | 19.75 ± 51.64 | < 0.05 |
| *Acinetobacter* | 0.06 ± 0.04 | 40.78±0.50 | > 0.05 | 0.31 ± 0.31 | 37.09 ± 4.81 | > 0.05 |
| *Rhodococcus* | 0.24 ± 0.11 | 38.79±2.71 | > 0.05 | 0.25 ± 0.19 | 38.89 ± 4.82 | < 0.05 |
| *Veillonella* | 0.06 ± 0.03 | 39.62±0.28 | < 0.05 | 0.02 ± 0.04 | 40.15 ± 0.50 | < 0.05 |
| *Aquabacterium* | 0.00 ± 0.00 | 41.62±0.09 | > 0.05 | 0.08 ± 0.15 | 40.13 ± 2.82 | < 0.05 |
| *Pantoea* | 0.01 ± 0.01 | 39.88±0.28 | < 0.05 | 0.08 ± 0.07 | 38.53 ± 1.33 | < 0.05 |
| *Peptostreptococcus* | 0.00 ± 0.00 | 39.88±0.03 | > 0.05 | 0.42 ± 0.15 | 29.89 ± 3.56 | > 0.05 |
